# Protective Effect of Optic Atrophy 1 on Cardiomyocyte Oxidative Stress: Roles of Mitophagy, Mitochondrial Fission, and MAPK/ERK Signaling

**DOI:** 10.1155/2021/3726885

**Published:** 2021-06-07

**Authors:** Yue Wang, Zhihua Han, Zuojun Xu, Junfeng Zhang

**Affiliations:** Department of Cardiology, Shanghai Ninth People's Hospital, Shanghai Jiaotong University School of Medicine, Shanghai 200011, China

## Abstract

Myocardial infarction is associated with oxidative stress and mitochondrial damage. However, the regulatory mechanisms underlying cardiomyocyte oxidative stress during myocardial infarction are not fully understood. In the present study, we explored the cardioprotective action of optic atrophy 1- (Opa1-) mediated mitochondrial autophagy (mitophagy) in oxidative stress-challenged cardiomyocytes, with a focus on mitochondrial homeostasis and the MAPK/ERK pathway. Our results demonstrated that overexpression of Opa1 in cultured rat H9C2 cardiomyocytes, a procedure that stimulates mitophagy, attenuates oxidative stress and increases cellular antioxidant capacity. Activation of Opa1-mediated mitophagy suppressed cardiomyocyte apoptosis by downregulating Bax, caspase-9, and caspase-12 and upregulating Bcl-2 and c-IAP. Using mitochondrial tracker staining and a reactive oxygen species indicator, our assays showed that Opa1-mediated mitophagy attenuated mitochondrial fission and reduced ROS production in cardiomyocytes. In addition, we found that inhibition of the MAPK/ERK pathway abolished the antioxidant action of Opa1-mediated mitophagy in these cells. Taken together, our data demonstrate that Opa1-mediated mitophagy protects cardiomyocytes against oxidative stress damage through inhibition of mitochondrial fission and activation of MAPK/ERK signaling. These findings reveal a critical role for Opa1 in the modulation of cardiomyocyte redox balance and suggest a potential target for the treatment of myocardial infarction.

## 1. Introduction

Oxidative stress in cardiomyocytes has been regarded as the primary pathological factor in many cardiovascular disorders including, but not limited to, diabetic cardiomyopathy, heart failure, myocardial hypertrophy, cardiac fibrosis, and dilated cardiomyopathy [1–3]. Particularly, recent studies have highlighted the important role of oxidative stress in the induction of myocardial infarction [2, 4, 5]. At the molecular level, oxidative stress induces the peroxidation of cellular membranes, including the mitochondrial membrane, the endoplasmic reticulum membrane, and the plasma membrane. Damage to the cellular membrane system disrupts cellular metabolism, accelerates cellular senescence, and promotes cell death [6, 7]. Although several antioxidative therapies have been developed to promote the recovery of cardiomyocyte function in cardiovascular disease, several questions remain regarding the upstream regulatory mechanisms controlling antioxidant responses in these cells [8–11].

Oxidative stress is primarily caused by excessive production and intracellular accumulation of reactive oxygen species (ROS) [12]. Since most cellular ROS are produced in the mitochondrion during oxidative phosphorylation [13], this organelle is widely recognized as a crucial target in the treatment of cardiovascular conditions [14–16]. Whereas physiological (low) ROS levels serve signaling functions and contribute to adaptive responses to hypoxia, excess ROS overwhelms the cells' antioxidant defenses and exacerbates mitochondrial ROS production to ultimately promote cell death [17]. Therefore, strategies aiming at protecting mitochondria and attenuating ROS production have great therapeutic potential in the management of cardiovascular disease [18]. Our previous study has reported a mitochondrial self-protection program involving mitochondrial autophagy (mitophagy) regulated by optic atrophy 1 (Opa1), a protein located at the inner mitochondrial membrane [19]. Moderate mitochondrial mitophagy promotes mitochondrial turnover, accelerates the recycling of damaged mitochondrial population, and blocks mitochondria-mediated cell death signaling [20–23]. However, the role of Opa1-related mitophagy in modulating cellular oxidative stress is not fully understood, especially in the setting of myocardial infarction.

Mitochondrial ROS production seems to be mainly affected by mitochondrial fission, a process necessary to control mitochondrial metabolism and oxidative phosphorylation [24, 25]. An increased mitochondrial population, as a result of mitochondrial fission, will accelerate glucose metabolism and therefore promote ATP production, an effect that is accompanied by enhanced ROS generation. Accordingly, inhibition of mitochondrial fission has been found to attenuate ROS levels in cardiomyocytes [26]. Although mitophagy serves as a mechanism to remove excess/fragmented organelles resulting from mitochondrial fission, it is unclear whether Opa1-mediated mitochondrial mitophagy exerts antioxidative effects through inhibition of mitochondrial fission.

The MAPK/ERK pathway has been reported as a main upstream regulator of mitochondrial fission [27]. Interestingly, there is also a close association between MAPK/ERK signaling and the activity of the cellular antioxidant system [28]. However, the relationship between Opa1-related mitochondrial mitophagy, the MAPK/ERK pathway, and mitochondrial fission remains unclear. Thus, in the present study, the hypothesis that Opa1-related mitophagy inhibits mitochondrial fission and oxidative stress through a mechanism involving the activation of the MAPK/ERK pathway was tested using control and Opa1-overexpressing H9C2 cardiomyocytes challenged with H_2_O_2_ to model myocardial infarction *in vitro*.

## 2. Materials and Methods

### 2.1. Cell Culture and Adenoviral Transduction

H9C2 cells were obtained from ATCC and cultured in DMEM/F12 supplemented with 10% FBS (Abcam, USA) at 37°C and 5% CO_2_ [29, 30]. Cells (2 × 10^5^) were seeded in six-well plates and transduced with an Opa1-encoding adenovirus (Ad-Opa1; VENDOR) at 37°C for 48 h using Lipofectamine® 2000 (Invitrogen, USA) [31]. To induce oxidative stress damage, H_2_O_2_ (0.3 mM) was added into the medium of H9C2 cardiomyocytes for 12 h [32].

### 2.2. CCK-8 Assay

Control and Ad-Opa1-transduced H9C2 cells were seeded onto 96-well plates and incubated with H_2_O_2_ (0.3 mM) for 12 h. A CCK-8 reagent was then added to each well and incubated for 4 h. Absorbance was detected at 490 nm [33].

### 2.3. Evaluation of Mitochondrial Morphology

H9C2 cells were seeded at a density of 6 × 10^5^ cells/well into 6-well plates, cultured for 24 h at 37°C in 5% CO_2_, and infected at an MOI of 50 with Ad-Opa1 for 48 h. Noninfected H9C2 cells were used as negative controls. After exposure to H_2_O_2_ (0.3 mM) or vehicle for 12 h, the cells were incubated in the dark for 30 min at 37°C in the presence of 4 *μ*M of MitoTracker™ Red [34]. Fluorescence microscopy (Olympus, Tokyo, Japan) was used to analyze mitochondrial morphology.

### 2.4. Assessment of Mitochondrial Membrane Potential

H9C2 cells were plated and transduced with Ad-Opa1 as described above, treated with vehicle or H_2_O_2_ (0.3 mM) for 12 h, and incubated in the dark for 20 min at 37°C in the presence of 1 *μ*L JC-1 in 1 mL of DMEM [35]. Fluorescence microscopy was then used to determine mitochondrial membrane potential [36].

### 2.5. ROS Assay

A cellular ROS red fluorescence assay kit (Cat. no. GMS10111.1; GENMED Scientifics, Inc., USA) was used to detect intracellular ROS. H9C2 cells (1 × 10^4^) were plated in 96-well plates and two days later exposed to H_2_O_2_ for 12 h. In some experiments, the cells were pretreated with FCCP, an activator of mitochondrial fission, or PD98059, a MAPK/ERK inhibitor, before being exposed to H_2_O_2_. The culture medium was aspirated, and 100 *μ*L of staining working solution was added according to the manufacturer's instructions [37]. The mixture was incubated at 37°C for 20 min in the dark and then washed with PBS three times. ROS fluorescence (*E*_*x*_/*E*_*m*_ = 540/590 nm) was measured on a microplate reader controlled by SkanIt software (Cat. no. N16699; Thermo Scientific, Inc., USA) [38, 39]. The results were presented as percentage fluorescence relative to the control group. Fluorescence microscopy was also performed in cells seeded on 6-well plates after exposure to H_2_O_2_ for 12 h. Following DAPI staining, an EVOS® FL Cell Imaging System (Life Technologies, USA) was used to conduct fluorescence imaging [40].

### 2.6. Evaluation of Cellular Antioxidant Activities

The activity of cellular antioxidant enzymes was measured through ELISA as previously described [41]. Colorimetric determinations of cellular antioxidant enzyme activities were performed using a Glutathione Reductase Assay Kit (Beyotime, China, Cat. No: S0055), a Total Superoxide Dismutase Assay Kit (Beyotime, Cat. No: S0101), and a Cellular Glutathione Peroxidase Assay Kit (Beyotime, Cat. No: S0056) [42].

### 2.7. TUNEL Staining

Cardiomyocyte apoptosis was determined using a One Step TUNEL apoptosis Assay Kit (Beyotime) according to the manufacturer's instructions [43]. After TUNEL labeling and DAPI counterstain, images were captured by fluorescence microscopy. Apoptosis was expressed as a percentage relative to the control group [44, 45].

### 2.8. Western Blot Analysis

Cells were harvested and lysed in RIPA buffer containing 1% protease inhibitor and 1% phosphatase inhibitor (Wako, USA). The lysates were mixed with 3x SDS sample buffer with 2-mercaptoethanol and boiled at 95°C for 5 min prior to SDS-PAGE [46]. Proteins were transferred to a PVDF membrane (Millipore, USA) and immunoblotted with the primary antibodies. The membranes were then incubated with anti-mouse or anti-rabbit HRP-conjugated secondary antibodies (1 : 1000, Bio-Rad, USA) and visualized using a chemiluminescence kit (Santa Cruz Biotechnology, USA) or SuperSignal West Femto Maximum Sensitivity Substrate (Thermo Fisher Scientific) [47].

### 2.9. RT-PCR

Total RNA was extracted with a TRIzol reagent (Invitrogen) and treated with DNase I to remove genomic DNA. Then, 500 ng of RNA was reversely transcribed into cDNA with the SuperScript IV First-Strand Synthesis System (Invitrogen) [48, 49] and quantitative real-time RT-PCR was performed using a Fast SYBR Green Master Mix (Fisher Scientific) according to the manufacturer's instructions and as described previously [50]. The PCR protocol consisted of 40 cycles of denaturation at 94°C for 15 s, annealing at 60°C for 15 s, and extension at 72°C for 1 minute, followed by a single, 7 min extension period at 72°C [51]. Expression levels of target genes were normalized to those of the endogenous glyceraldehyde phosphate dehydrogenase (GAPDH) [52].

### 2.10. Statistics

Data are presented as mean ± SEM. Each *in vitro* experiment was performed at least three times. Significance (*p* < 0.05) was determined via Student's *t*-test for comparisons between two groups and via two-way ANOVA for comparisons between three or more groups.

## 3. Results

### 3.1. Opa1-Mediated Mitophagy Attenuates ROS Production and Mitochondrial Dysfunction

**Figure 1:**
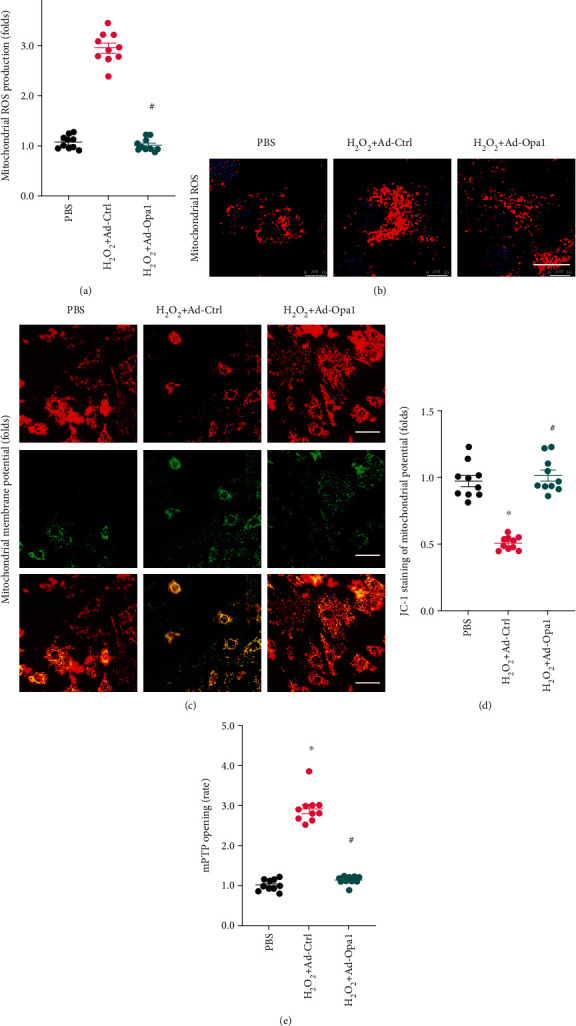
Opa1-mediated mitophagy attenuates mitochondrial ROS production and reduces mitochondrial dysfunction. Control (nontransduced) and Ad-Opa1-transduced H9C2 cardiomyocytes were treated with 0.3 mM H_2_O_2_ for 12 h. (a, b) Fluorescent detection of ROS production. Bar: 15 *μ*m. (c, d) Assessment of mitochondrial membrane potential via JC-1 staining. Bar: 40 *μ*m. (e) Results of the mPTP opening assay. ^∗^*p* < 0.05 vs. control group, ^#^*p* < 0.05 vs. H_2_O_2_+Ad-Ctrl group.

To investigate the role of Opa1-related mitophagy on ROS production and mitochondrial dysfunction, cultured H9C2 cardiomyocytes were transduced with an adenoviral vector encoding Opa1 (Ad-Opa1) before being exposed to H_2_O_2_ (0.3 mM) to induce an oxidative stress microenvironment. Nontransduced cells were used as the control. As shown in Figures 1(a) and 1(b), the production of mitochondrial ROS was significantly increased in nontransduced cardiomyocytes and significantly inhibited in cells overexpressing Opa1. Since mitochondrial ROS overproduction is associated with oxidative stress and mitochondrial and cellular dysfunction, we next evaluated the functionality of mitochondria in both control and Opa1-transduced cardiomyocytes. As shown in Figures 1(c) and 1(d), mitochondrial membrane potential was reduced by H_2_O_2_ exposure in control cells but remained largely unaffected after Opa1 overexpression. Excessive mitochondrial damage is associated with mitochondria-dependent cardiomyocyte death. Using ELISA, we found that the rate of mitochondrial permeability transition pore (mPTP) opening, an early marker of cardiomyocyte death following ischemia-reperfusion injury, was significantly elevated in control cardiomyocytes treated with H_2_O_2_. However, overexpression of Opa1 was able to block mPTP opening (Figure 1(e)). These data demonstrate that Opa1-mediated mitophagy suppresses mitochondrial ROS production and maintains mitochondrial function under an oxidative stress microenvironment.

### 3.2. Opa1-Induced Mitophagy Increases the Activity of Cellular Antioxidant Enzymes

**Figure 2:**
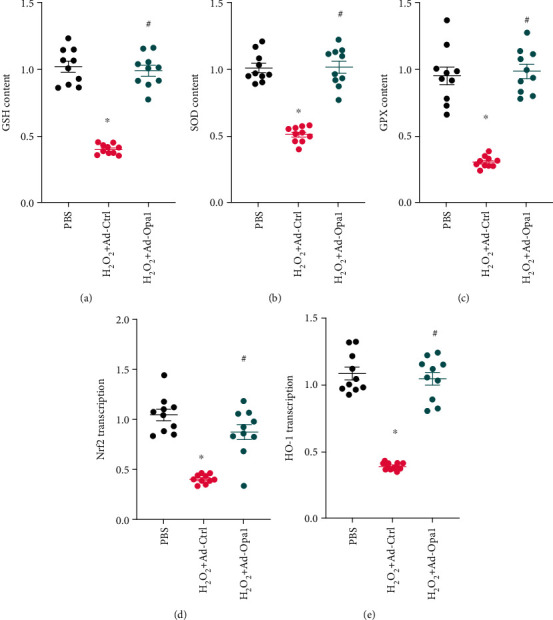
Opa1-mediated mitophagy increases the activity of cellular antioxidant enzymes. Control (nontransduced) and Ad-Opa1-transduced H9C2 cardiomyocytes were treated with 0.3 mM H_2_O_2_ for 12 h. (a–c) Colorimetric determination of GSH, SOD, and GPX activities. (d, e) Results of qPCR assays to analyze the transcriptional profiles of Nrf2 and HO-1. ^∗^*p* < 0.05 vs. control group, ^#^*p* < 0.05 vs. H_2_O_2_+Ad-Ctrl group.

Since mitochondrial oxidative stress can also result from decreased antioxidant capacity, we asked whether Opa1-induced mitophagy would also regulate the activity of antioxidant enzymes. We found that the levels of GSH, SOD, and GPX were significantly reduced in H_2_O_2_-treated cardiomyocytes. Interestingly, these changes were reversed by Opa1 overexpression (Figures 2(a)–2(c)). Since the activity of cellular antioxidant enzymes is primarily regulated at the transcriptional level, we assessed the impact of Opa1 overexpression on the expression of two key transcription factors, namely, Nrf2 and HO-1, governing the expression of antioxidant enzymes. Results of qPCR analysis demonstrated that Nrf2 and HO-1 mRNA levels were markedly downregulated in cardiomyocytes treated with H_2_O_2_ but upregulated instead after Opa1 overexpression (Figures 2(d) and 2(e)). These data indicate that Opa1-mediated mitophagy enhances the antioxidant potential of cardiomyocytes through upregulation of the transcription of HO-1 and Nrf2.

### 3.3. Opa1-Mediated Mitophagy Sustains Cardiomyocyte Viability under Oxidative Stress Conditions

**Figure 3:**
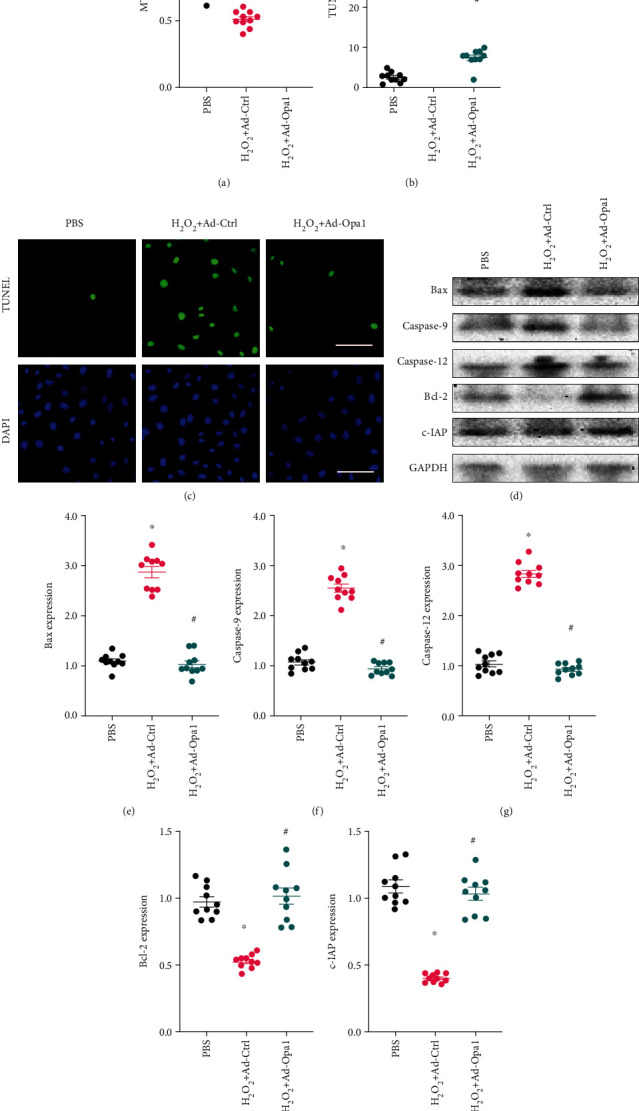
Opa1-mediated mitophagy sustains cardiomyocyte viability under oxidative stress. Control (nontransduced) and Ad-Opa1-transduced H9C2 cardiomyocytes were exposed to 0.3 mM H_2_O_2_ for 12 h. (a) Analysis of cardiomyocyte viability using the CCK-8 assay. (b, c) Assessment of apoptosis by TUNEL assay. Bar: 90 *μ*m. (d–i) Western blotting analysis of Bax, caspase-9, caspase-12, Bcl-2, and c-IAP expression. ^∗^*p* < 0.05 vs. control group, ^#^*p* < 0.05 vs. H_2_O_2_+Ad-Ctrl group.

Under oxidative stress conditions, impaired mitochondrial function and limited antioxidant capacity reduce the viability of cardiomyocytes by activating cell death pathways. Results of CCK-8 assays demonstrated that the viability of H9C2 cells was significantly reduced in response to H_2_O_2_ treatment. In contrast, cell viability was significantly rescued by Opa1 overexpression (Figure 3(a)). This finding was further analyzed using TUNEL staining. As shown in Figures 3(b) and 3(c), apoptosis was significantly promoted after exposure to H_2_O_2_. In turn, induction of mitophagy via Opa1 overexpression markedly decreased the number of apoptotic cardiomyocytes. To investigate the molecular basis underlying Opa1-mediated antiapoptotic action, western blots were used to analyze changes in cell death-related protein expression. As shown in Figures 3(d)–3(i), a significant increase in Bax, caspase-9, and caspase-12 expression, paralleled by downregulation of Bcl-2 and c-IAP expression, was observed in cardiomyocytes treated with H_2_O_2_. Interestingly, after overexpression of Opa1, the expression of proapoptotic proteins was reduced, while the levels of antiapoptotic proteins were restored to near normal levels. These data indicate that the reduction in cardiomyocyte viability mediated by H_2_O_2_ exposure can be reversed by Opa1-induced mitophagy.

### 3.4. Opa1-Mediated Mitophagy Inhibits Mitochondrial Fission

**Figure 4:**
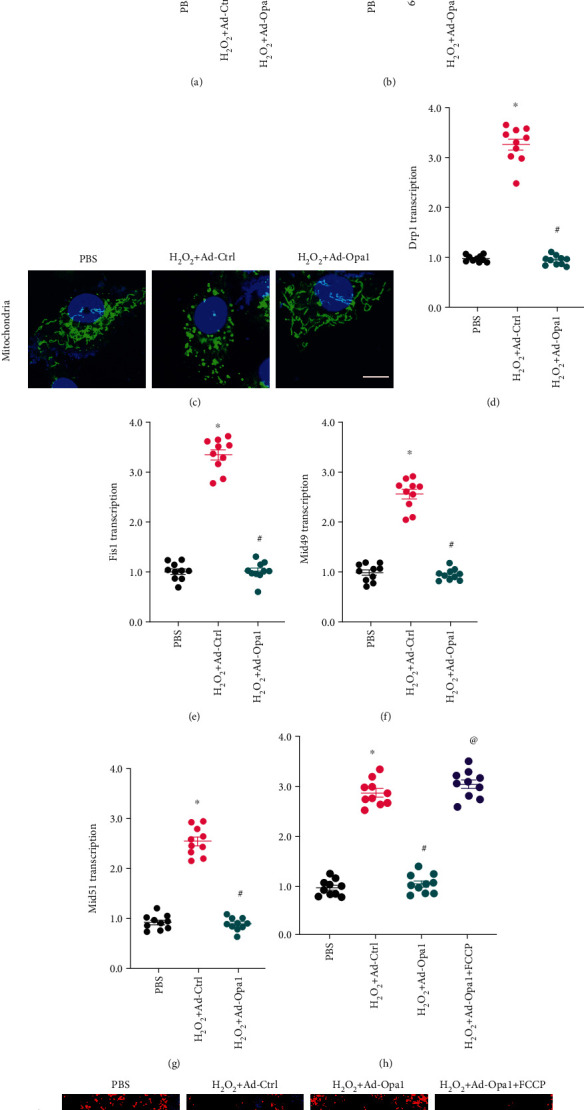
Opa1-mediated mitophagy inhibits mitochondrial fission. Control (nontransduced) and Ad-Opa1-transduced H9C2 cardiomyocytes were exposed to 0.3 mM H_2_O_2_ for 12 h. (a–c) Evaluation of mitochondrial morphology by MitoTracker Red staining. Bar:20 *μ*m. (d–g) Results of qPCR assays to analyze the transcriptional profiles of Drp1, Fis1, Mid49, and Mid51. (h, i) Fluorescent detection of ROS production in H9C2 cells pretreated with or without the mitochondrial fission activator FCCP. Bar: 25 *μ*m. ^∗^*p* < 0.05 vs. control group, ^#^*p* < 0.05 vs. H_2_O_2_+Ad-Ctrl group, ^@^*p* < 0.05 vs. H_2_O_2_+Ad-Opa1 group.

Recent studies have reported that mitochondrial fission is the primary trigger of mitochondrial ROS overproduction through accelerated glucose metabolism. To evaluate whether Opa1-mediated mitophagy can attenuate abnormal mitochondrial fission under oxidative stress conditions, mitochondrial morphology was first examined in cultured H9C2 cardiomyocytes using MitoTracker staining. As shown in Figures 4(a)–4(c), H_2_O_2_ treatment elicited substantial mitochondrial fragmentation, evidenced by an increase in the organelles' average length and number. In contrast, both these variables were significantly normalized in Ad-Opa1-transduced cardiomyocytes. To provide more evidence to support the regulatory role played by Opa1-related mitophagy on mitochondrial fission, qPCR was performed to analyze transcriptional levels of fission-related proteins. As shown in Figures 4(d)–4(g), compared to the control group, the expression of Drp1, Fis1, Mid49, and Mid51 was significantly increased in cardiomyocytes treated with H_2_O_2_. However, after transduction with Opa1 to stimulate mitophagy, Drp1, Fis1, Mid49, and Mid51 mRNA levels were markedly upregulated. Taken together, these results demonstrated that Opa1-related mitophagy inhibits mitochondrial fission in H_2_O_2_-treated cardiomyocytes.

To evaluate whether Opa1 represses mitochondrial ROS through inactivation of mitochondrial fission, before being exposed to H_2_O_2_, Opa1-overexpressing cardiomyocytes were pretreated with FCCP, an activator of mitochondrial fission. As shown in Figures 4(h) and 4(i), FCCP abolished the inhibition of ROS production elicited by Opa1 overexpression. Overall, our data illustrated that Opa1-mediated mitochondrial ROS suppression is attributable to decreased mitochondrial fission.

### 3.5. Opa1-Mediated Mitophagy Activates the MAPK/ERK Signaling Pathway

**Figure 5:**
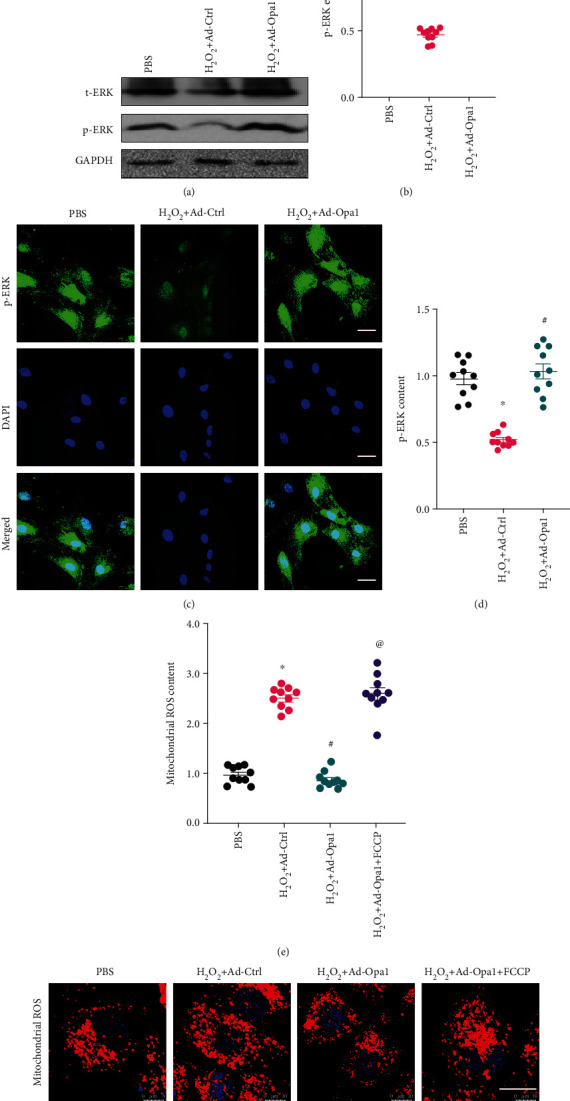
Opa1-mediated mitophagy activates the MAPK/ERK signaling pathway. Control (nontransduced) and Ad-Opa1-transduced H9C2 cardiomyocytes were treated with 0.3 mM H_2_O_2_ for 12 h. (a, b) Western blotting analysis of ERK and p-ERK expression. (c, d) Analysis of ERK and p-ERK expression through immunofluorescence. Bar: 75 *μ*m. (e, f) Fluorescent detection of ROS production in cells pretreated with the MAPK/ERK inhibitor PD98059. Bar: 25 *μ*m. ^∗^*p* < 0.05 vs. control group, ^#^*p* < 0.05 vs. H_2_O_2_+Ad-Ctrl group, ^@^*p* < 0.05 vs. H_2_O_2_+Ad-Opa1 group.

Since the MAPK/ERK pathway has been reported to be a regulator of mitochondrial ROS production, we wanted to know whether Opa1-mediated mitophagy restricts ROS production in cardiomyocytes by activating MAPK/ERK signaling. Western blot results demonstrated that the expression of p-ERK was significantly downregulated in cardiomyocytes treated with H_2_O_2_, and this effect was inhibited upon Ad-Opa1 transduction (Figures 5(a) and 5(b)). As shown in Figures 5(c) and 5(d), similar results were observed after p-ERK immunofluorescence. Based on the above data suggesting that Opa1-mediated mitophagy is an upstream activator of the MAPK/ERK pathway, the involvement of MAPK/ERK activation in Opa1-mediated mitochondrial ROS suppression was evaluated using PD98059, a MAPK/ERK signaling inhibitor. As shown in Figures 5(e) and 5(f), the inhibition exerted by Opa1 overexpression on H_2_O_2_-induced mitochondrial ROS production was significantly suppressed after PD98059 incubation. Overall, these data suggest that Opa1-mediated mitophagy attenuates mitochondrial ROS production by activating the MAPK/ERK signaling pathway.

## 4. Discussion

Although timely reperfusion therapies have been introduced for clinical treatment of myocardial infarction, organ recovery is hampered by cardiomyocyte death resulting from these treatments. Oxidative stress has been identified as a primary pathological factor mediating cardiomyocyte dysfunction and death during myocardial infarction and after reperfusion therapy. Although several antioxidant therapies have been developed for the management of patients with myocardial infarction, the mechanisms that regulate antioxidant responses in the postinfarcted heart remain unclear. In the present study, we identified Opa1-mediated mitophagy as a negative regulator of cardiomyocyte oxidative stress by preventing mitochondrial fission and activating the MAPK/ERK pathway. To our knowledge, this is the first evidence supporting the relationship between Opa1-mediated mitophagy and cardiomyocyte oxidative stress in an *in vitro* model of myocardial infarction. Therefore, our findings suggest a promising new target for the treatment of this condition and the pathological manifestations of ischemia-reperfusion.

Cardiomyocytes contain numerous mitochondria, which metabolize glucose to produce the large amounts of ATP required to sustain cardiac function. Accordingly, mitochondrial damage induces adverse metabolic reprogramming, resulting in cardiomyocyte dysfunction and death [53]. Since mitochondria also play a fundamental role in transmitting and amplifying proapoptotic signals during ischemic insults, these organelles represent a primary target for the treatment of myocardial infarction [54, 55]. Indeed, intensive research has revealed potential therapeutic avenues targeting mitochondrial dysfunction in cardiac cells. For example, thioredoxin has been found to sustain mitochondrial morphology and thus attenuate myocardial infarction through redox-dependent activation of CREB signaling [56]. Improvement in mitochondrial function during myocardial infarction through stabilization of the expression of Mzb1 was reported to attenuate the inflammatory response and delay cardiac fibrosis [57]. Inhibition of mitochondrial ROS production and thus attenuation of oxidative stress has been found to enhance cardiomyocyte ATP supply and sustain contractile function during hypoxia/reoxygenation stress [58–60]. Blockade of mitochondria-mediated cell death through deletion of PGAM5 was shown to improve mitochondrial quality control and reduce myocardial infarction size [61]. In turn, reduction in mitochondrial calcium overload through overexpression of SERCA was reported to improve myocardial perfusion and metabolism [62].

In the present study, mitochondrial damage induced by oxidative stress is featured by decreased mitochondrial membrane potential, increased mitochondrial ROS production, and elevated expression of mitochondria-related proapoptotic proteins. Importantly, we further show that enhanced mitophagy mediated by Opa1 overexpression is able to attenuate mitochondrial damage in oxidative stress-challenged cardiomyocytes. The protective mechanism afforded by Opa1-mediated mitophagy involves inhibition of mitochondrial fission and activation of the MAPK/ERK pathway. The protective role of mitophagy on mitochondrial homeostasis and cardiomyocyte viability has been widely reported [63–65]. For example, irisin treatment significantly activates Opa1-mediated mitophagy and thus inhibits cardiomyocyte mitochondrial apoptosis following myocardial infarction [66–68]. In turn, activation of Fundc1 leading to mitophagy sustains mitochondrial metabolism and promotes mitochondrial biogenesis in the ischemic myocardium [69, 70]. These findings are thus consistent with our results. Interestingly, our data further illustrate the regulatory role of Opa1-mediated mitophagy in the suppression of mitochondrial fission, by removing fragmentated mitochondria via lysosomal degradation.

Finally, we also report that Opa1-mediated mitophagy is an upstream trigger of the MAPK/ERK pathway in cardiomyocytes. This is in line with previous studies, conducted in aged parkinsonian mice [71–73], in a murine model of sleep apnea [74], and in a model of doxorubicin-induced cardiotoxicity [75] that highlighted the relationship between mitophagy and the ERK signaling pathway. Considering the necessary role played by ERK in regulating cardiomyocyte metabolism and ATP generation, mitophagy-induced ERK activation appears as a critical mechanism to support mitochondrial metabolism and oxidative phosphorylation under hypoxic conditions.

Taken together, our results demonstrated that Opa1-mediated mitophagy functions as a protective program against oxidative stress in cardiomyocytes through two different mechanisms: one involved in the inhibition of mitochondrial fission and the other driven by activation of the MAPK/ERK pathway. However, our study has many limitations that need to be addressed. First, we employed only *in vitro* experiments to elucidate the protective effects of Opa1-mediated mitophagy in oxidative stress-challenged cardiomyocytes. Thus, animal experiments are necessary to validate our findings *in vivo* [76]. Also, although our study stresses the functional importance of Opa1-mediated mitophagy in cardiomyocyte survival and function, further research on its protective mechanisms are limited by the lack of drugs to specifically activate Opa1 in cardiomyocytes [77].

## Data Availability

The analyzed data sets generated during the present study are available from the corresponding author on reasonable request.
